# Notices

**Published:** 1864-09

**Authors:** 


### NOTICES.

On the first of March last the subscription price to the Journal was raised to one dollar and a half a year; those who have sent their names since with but one dollar, are requested to send fifty cents in addition if they desire to have the Journal sent to them the remainder of the year.

To Physicians.—Braithwaite's Retrospect is published twice a year, giving a full and succinct account of all that is new in medicine and surgery throughout the world for the preceding six months. $2.50 a year in advance, or $1.50 each. Wm. A. Townsend, 55 Walker street, New-York. Part 49, for July, 1864, 300 pages 8vo, contains 124 articles, with a full and judicious synopsis and an extended alphabetical index of every subject treated. If any physician or surgeon takes but one medical publication, he should take the Retrospect, for it contains in a concentrated form an amount of medical information equal to any other half-dozen publications.

Harper's Weekly, $3 a year, and the Monthly at the same price, both for five dollars, maintain their popularity among all classes of readers.

The Atlantic Monthly, $3 a year, Boston, has just entered a new volume. It has a corps of writers embracing the finest talent in the land, hence its increasing popularity. But we are constrained to say we do not consider it a true friend to the Christian religion; it seems to be, but every now and then its best contributors give it a malignant stab—Holmes, Hawthorne, Emerson, and some others. We wish it were otherwise, for with that exception it is by far the most ably conducted literary monthly either in Great Britain or America.

The following beautiful volumes are among the issues of the American Tract Society, 28 Cornhill, Boston, and 13 Bible House, New-York: Trevor's Ancient Egypt, Walter Lightfoot's Pictures, New Stories from an Old Book, Progress, or the Sequel to Jerry and his Friend, by Alice A. Dodge, The Gospel among the Caffirs, or the Story of Rev. Mr. Moffatt and his labors in South-Africa, a most interesting history, and as instructive as Trevor's Ancient Egypt. Then there is a beautiful 24mo, Stories for Little Ones, second series, containing over twenty narrations for young children. "Our Birds," by Mrs. Fanny J. Burge Smith, contains beautiful engravings of a dozen of the sweetest birds of the world, the jay, martin, woodpecker, oriole, bobolink, sparrow, mocking, humming, and cat-birds. Missions and Martyrs in Madagascar—no man can feel as he ought, until he reads this book, the inexpressible happiness of being permitted in peace and quietness to live in a land of religious liberty. "Christian Home Life," a book of examples and principles, 13 chapters on Bible and Home Life, Piety at Home, Home Happiness, Teaching and Training, Formation of Character, Personal Habits, Social Habits, Child Piety, Family Worship, Sabbath at Home, Social Intercourse, Breaking Up of Home, The Eternal Home. These headings show the richness of the book. What thousands will want to read the "Autographs of Mrs. Sherwood," 441 pp. 12mo, with extracts from Mr. Sherwood's journal during his imprisonment in France and residence in India, abridged from the London edition.

Going Home.—Only eleven Revolutionary pensioners "still live." The twelfth was Rev. Daniel Waldo, just died, August, 1864, having lived nearly a hundred and two years. Within a year he has preached several times twice on the Sabbath; his disease was a throat affection.

### NOTICES.

The Family Treasure, monthly, $2, Pittsburgh, Pa., by David McKinney, D.D., and I. N. McKinney, is well worthy the patronage of all moral and religious families, and such as aim at a high moral culture. It is for the family, and is edited with sound judgment and a wise discrimination. Let every religious family try it a year. It is devoted to "Christian doctrine, science, history, biography, and evangelical literature." May it fill the want of page 178.

Economical eating bids fair to be the rage now. We have a tract on that subject in the present number. The white bean item is worthy of special note; we have spoken in its praise. We generally aim to under-state; but in the effort in this case we did not do the beans equal and impartial justice. We said they went farther than roast beef. This was after having made the trial at our own table; our family of eight will consume four pounds of roast beef, butcher's weight, a day. We called for a pint of white beans, had them weighed, a little over one pound, paid seven cents for them, and had a dish of "pork and beans," so the cook said. It was so unusual a sight, our little Alice inquired with singular earnestness, opening her eyes to the dimensions of young saucers, and pointing to the dish: "Mother, what *is* that?" In order to give the dish a fair chance, there was no other meat on the table. And we are bound to speak the praise of beans in the way of economy; this one pound certainly did go farther, costing but seven cents, than a four-pound roasting-piece of beef. The beans went a week. Therefore, beans "*is*" economical, very. *Quod erat dem.* But for all that, the white beans can't hold a candle to the cheap bread we have lauded as unstintedly as Orange Judd of the American Agriculturist, which first gave publicity to the receipt. Our own tastes are not very acute; we had to ask the waiter this morning whether we were drinking coffee or tea; ordinarily we know by casting our eye at the head of the table and noticing the shape of the beverage-holder; but wifey being out of town, there was no urn on the table. But as to the famous corn-bread; in order to make a fair experiment, we allowed no other kind to come on the table, so that wife, children, or servants had to eat that or none. Well, the result of the operation is, that the entire family suddenly lost all appetite for bread, and five pounds of corn-meal lasted eight persons seven days, by which time the experiments were ended, the show was over, the school out, and "as you were" is the order of the day, save and excepting the *bulk* of my purse, length and weight being now obsolete terms in that connection.

P. S.—N. B. We stop the press to say, that since writing the foregoing the thought occurred that the family may have found some substitute for bread; and on looking at the grocer's "pass-book," there are frequent items of "soda crackers." Hence, instead of feeding our family on corn-bread at five cents, or even home-made wheat bread at eight cents, we have been paying fifteen cents a pound for "soda crackers," eaten at lunch, when we are down-town. Wonder after all, if a good many "experiments" of world-wide publicity, are not about as truthful, *practically*, as ours with white beans and corn-meal. So our readers must remember, that a thing may be literally true, but for all practical purposes false. Meanwhile we will rest in the conclusion, that the virtuous and patriotic public will economize, only when there is no beef or flour to be purchased, or they have no money to buy.

Special attention is invited to the advertisement of those sterling and enterprising business men, Messrs. Scott & Baldwin, of 505 Broadway. See their principles of doing business in our July number, and which have been copied in the magazines and newspapers for their excellence, and which ought to be practiced by every business man in the nation.

Sweeping Conscription of every copy that could be found in the city, for the army, of our condensed edition of Soldier Health, 32 pp., five cents, six by mail. It is the most urgently demanded book yet written for the soldiers. Who will send in thirty dollars to hand over one thousand copies to the Christian Commission, or to supply a named regiment?

